# Public health round-up

**DOI:** 10.2471/BLT.24.011124

**Published:** 2024-11-01

**War engulfs the Eastern Mediterranean Region**

The rubble of a building destroyed in an Israeli airstrike on the southern suburbs of Beirut, Lebanon on 20 September. The escalation of hostilities in that country marked a significant development in the conflict that has engulfed the Eastern Mediterranean Region since 7 October 2023.

**Figure:**
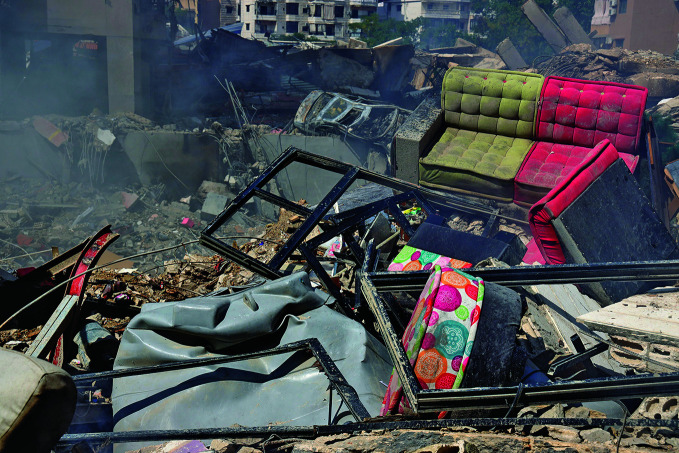
UNICEF/Dar al Mussawir/Ramzi Haidar

## Conflict in the Eastern Mediterranean Region

The escalation of hostilities in Lebanon marked a widening of the conflict that has engulfed the Eastern Mediterranean Region since 7 October 2023. In a 3 October media briefing, World Health Organization (WHO) Director-General Tedros Adhanom Ghebreyesus reported that the conflict had claimed the lives of more than 1500 people in Israel, nearly 42 000 in Gaza, and over 700 in the West Bank. In Gaza, more than 10 000 people have also been reported missing, while 1.9 million people have been displaced. Additionally, 101 hostages taken from Israel remained in Gaza.

In Lebanon, over 1600 people had been killed and more than 1 million displaced, including 350 000 forced to live in shelters and 160 000 who had crossed into the Syrian Arab Republic. Syria’s government, the United Nations and other partners are working to manage the new wave of refugees.

Health facilities have also been targeted, including the 37 health facilities that were forced to close in Lebanon, where 28 health workers were killed in a period of 24 hours, and many others fled their posts due to bombardments.

Despite these challenges, health and humanitarian workers continued their work in dangerous conditions and with limited supplies. WHO urged partners to facilitate flights to deliver life-saving supplies to Lebanon and called for de-escalation of the conflict, the protection of health-care facilities and the securing of access routes for humanitarian aid.

https://bit.ly/4f3nyy5

## Rwanda Marburg outbreak

The Rwanda Ministry of Health confirmed an outbreak of Marburg virus disease (MVD) on 27 September, the first time the disease has been reported in the country. As of 3 October, 36 people were confirmed to have been infected, including 11 people who died in seven of the country's 30 districts.

As of the same date, 25 patients were in isolation receiving care, while contact tracing efforts were ongoing, with 300 individuals being monitored. Over 70% of the confirmed infections were of health workers from two facilities in the capital, Kigali.

The Rwandan government coordinated response efforts with the support of WHO and other partners. WHO deployed experts to assist in areas such as infection prevention, clinical care, logistics and technical research to understand transmission and prevent more cases.

https://bit.ly/3BJrdCH

https://bit.ly/4f3nyy5

## mpox diagnostic listing

WHO listed the first mpox in vitro diagnostic test under its Emergency Use Listing procedure, an important step in improving global access to mpox testing.

Announced on 3 October, the approval for emergency use of the Alinity m MPXV assay will support the expansion of diagnostic capacity in countries facing mpox outbreaks.

Limited testing capacity and delays in confirming mpox cases persist in the African Region, contributing to the continued spread of the virus. In 2024, over 30 000 suspected cases have been reported across the region, with the highest numbers in the Democratic Republic of the Congo, Burundi and Nigeria. In the Democratic Republic of the Congo, it estimated that just over a third of people with suspected infections have been tested this year.

https://bit.ly/3Utmpbh

## Evolving pandemic risk

The nature of pandemic risk is evolving, driven by increased biosecurity threats, rapid technological innovation, misinformation, intensive farming practices and entrenched social and economic inequity.

This is among the conclusions of a new report published by the Global Preparedness Monitoring Board (GPMB). Co-convened by WHO and the World Bank, the GPMB is tasked with tracking the drivers of pandemic risk and overseeing global preparedness.

Launched at the 15th World Health Summit in Berlin, Germany on 14 October, *The changing face of pandemic risk* outlines 15 key drivers of pandemic risk and provides a roadmap for the global response. It calls on leaders to adapt, protect and connect to ensure that efforts to prevent pandemics today are strong and flexible enough to respond to the next pandemic.

https://bit.ly/4eKCkKi

## Tackling antimicrobial resistance

Heads of state and government representatives issued a political declaration on antimicrobial resistance (AMR) at the 79th United Nations General Assembly held in New York between 22 and 27 September.

The declaration includes a commitment to reducing the estimated 4.95 million human deaths associated with AMR annually by 10% by 2030, and to ensuring that 80% of countries have access to testing for AMR infections by that date.

The declaration also contained a commitment on water, sanitation and hygiene and 100 million United States dollars (US$) in catalytic funding to boost national financing for national action plans on AMR.

In related news, WHO released a report on the potential role of vaccines in reducing AMR on 10 October. The report outlines the importance of vaccines as a crucial tool in preventing infections and curbing the spread of resistant strains, and argues that investment in vaccines could reduce antibiotic use, avert AMR-associated deaths and save money spent treating AMR infections.

https://bit.ly/3No8Vto

https://bit.ly/3U0XM5u

## WHO arboviral disease plan

WHO launched a plan designed to support efforts to reduce the burden of disease, suffering and deaths from dengue and other mosquito-borne diseases such as Zika and chikungunya.

Launched on 3 October, the *Global strategic preparedness, readiness and response plan (SPRP) to tackle dengue and other *Aedes*-borne arboviruses* outlines priority actions to control transmission and offers recommendations to affected countries across various sectors.

An estimated four billion people are at risk of infection from arboviruses around the world, a number projected to increase to 5 billion by 2050. Dengue cases have surged across all six WHO regions, the number of cases roughly doubling each year since 2021, with over 12.3 million cases as of the end of August this year – almost double the 6.5 million cases reported in all of 2023.

https://bit.ly/3Y4b1Dl

## Neglected tropical disease progress

WHO congratulated Brazil for having eliminated lymphatic filariasis as a public health problem (elimination is defined as decreasing the prevalence and transmission of the causal parasite and managing cases). Commonly known as elephantiasis, lymphatic filariasis is caused by filarial parasites which are transmitted to humans through mosquitoes. Infection is usually acquired in childhood and causes hidden damage to the lymphatic system.

With the 1 October announcement, Brazil joined 19 other countries and territories validated by WHO for having eliminated the disease. In 2023, 657 million people in 39 countries and territories were living in areas that continue to require preventive chemotherapy to stop the spread of lymphatic filariasis infection.

In related news, WHO validated the elimination of trachoma as a public health problem (reducing the prevalence of trachoma to levels where it no longer poses a significant health risk to the population) in Pakistan, making it the 19th country globally to reach this historic milestone. Trachoma is caused by infection by the *Chlamydia trachomatis* bacterium and is the leading infectious cause of blindness globally.

https://bit.ly/3zWwFle

https://bit.ly/4eXXWm7

## Clinical trial guidance

WHO released guidance to improve the design, conduct and oversight of clinical trials in countries of all income levels. Released on 25 September, the guidance aims to support stronger country-led research and development ecosystems. For the first time, the guidance includes recommendations for national health authorities, regulatory authorities, funders and others on how they can best facilitate clinical trials to generate evidence on health interventions.

https://bit.ly/483vtsG

## WHO prequalifies new human papillomavirus vaccine

WHO announced that a fourth WHO-prequalified human papillomavirus (HPV) vaccine product, Cecolin® has been confirmed for use in a single-dose schedule. Announced on 4 October, the decision was made based on new product data and will significantly contribute to a sustainable supply of HPV vaccines – allowing more young women to be reached with the vaccines that prevent cervical cancer.

https://bit.ly/4eXfFKo

**Cover photo**

A man considers the remains of buildings destroyed by Israeli military airstrikes in southern Beirut, Lebanon in September 2024.

**Figure:**
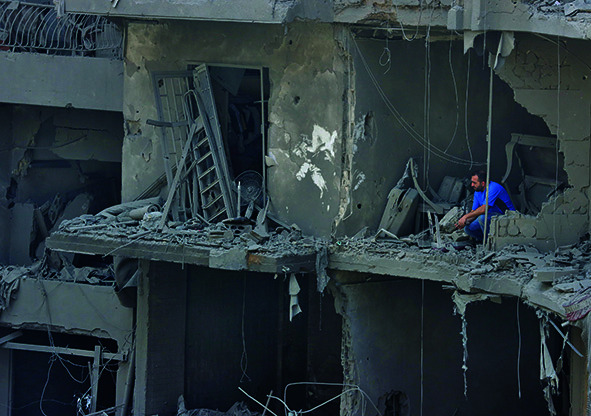
UNICEF/Dar al Mussawir/Ramzi Haidar

**Looking ahead**

7–8 November. Ministerial Conference on Ending Violence Against Children. Bogota, Colombia. https://bit.ly/3YjDHJV

13–14 November. The World Innovation Summit for Health. Doha National Convention Centre, Doha, Qatar. https://bit.ly/3Y5dL3H

26–29 November. WHO global oral health meeting. Bangkok, Thailand. https://bit.ly/4fiQEcX

